# Radiomics-Informed Modeling for Transcranial Ultrasound Stimulation: Age Matters

**DOI:** 10.3389/fnins.2022.935283

**Published:** 2022-06-15

**Authors:** Hanna Lu

**Affiliations:** ^1^Department of Psychiatry, The Chinese University of Hong Kong, Hong Kong, Hong Kong SAR, China; ^2^Centre for Neuromodulation and Rehabilitation, The Affiliated Brain Hospital of Guangzhou Medical University, Guangzhou, China; ^3^The Affiliated Brain Hospital of Guangzhou Medical University, Guangzhou, China

**Keywords:** transcranial ultrasound stimulation, MRI, radiomics, scalp-to-cortex distance, transcranial model, age-specific, cortical thickness

## Introduction

Transcranial ultrasound stimulation (TUS) is emerging as a powerful, non-invasive neurotechnology for focal brain stimulation using low intensity focused ultrasound delivered through the scalp/skull to initiate the modulation of neuronal excitability in the targeted brain structures (Fomenko et al., 2020). With the advantages of higher spatial resolution and deeper penetration depth, TUS is increasingly employed as a potential alternative approach for overcoming the disadvantages of the currently used non-invasive modalities of brain stimulation. Different from transcranial magnetic stimulation (TMS) and transcranial current stimulation (tCS), the mechanism of TUS is the mechanical interaction between ultrasound waves and neuronal membranes, which can modulate the mechanosensitive voltage-gated ion channels or neurotransmitter receptors and achieve therapeutic goals (Tyler et al., 2008; Di Biase et al., 2019; Liao et al., 2019). For instance, evidence that a frequency-specific acoustic wave can be converted into an effective stimulus for a neuron has been observed in a quantitative radiation force model (Menz et al., 2019). In the last decade, TUS has shown to modulate the activities in retina (Menz et al., 2019), cortical and subcortical structures, resulting in electrophysiological and behavioral changes in mice (Hou et al., 2021), primates (Verhagen et al., 2019) and human (Ai et al., 2018). Among the published TUS studies, the mean chronological age derived from the human participants is around age 30 (see Supplementary Table 1), which conceivably limits the applications of TUS in children and elderly.

Using individual medical images as guidance, the accuracy of localizing the treatment targets during TUS has improved to millimeter (mm) scale, whereas the stimulation-related parameters and TUS-induced effect still vary between individuals, particularly in the individuals with age-related brain diseases, such as neurodevelopmental disorders and neurodegenerative diseases. Among the parameters that determine the heterogeneity of TUS-induced effect, the morphometric features of treatment target have been highlighted as the stimulation-specific factors (Polanía et al., 2018).

## Why Age-Specific Transcranial Models Important?

When TUS sonication is administered transcranially, the estimation of the focal area based on the stimulation is critical for precisely targeting the region of interest with effective energy intensity in the brain structure due to acoustic attenuation and refraction at skull and cerebrospinal fluid (CSF). In the consideration of the heterogeneity in skulls and brains, there is a pressing need to develop effective and advanced models of TUS, both as tool to investigate the transcranial features and as potential guidance for age-related brain diseases, such as pediatric disease (Janwadkar et al., 2022), neurodegenerative disease (Lipsman et al., 2018; Martínez-Fernández et al., 2018; Jeong et al., 2021). Except for sonication parameters (i.e., viscosity, stimulation frequency), I would like to highlight the parameters of the transcranial features embedded in the distance from scalp to cortex, including scalp, skull, CSF and cortex. Different from TMS and tCS, skull thickness, rather than CSF, can greatly influence the penetration depth (i.e., peak intracranial pressure) of TUS (Robertson et al., 2017; Guo et al., 2021; Riis et al., 2022).

**Figure 1:**
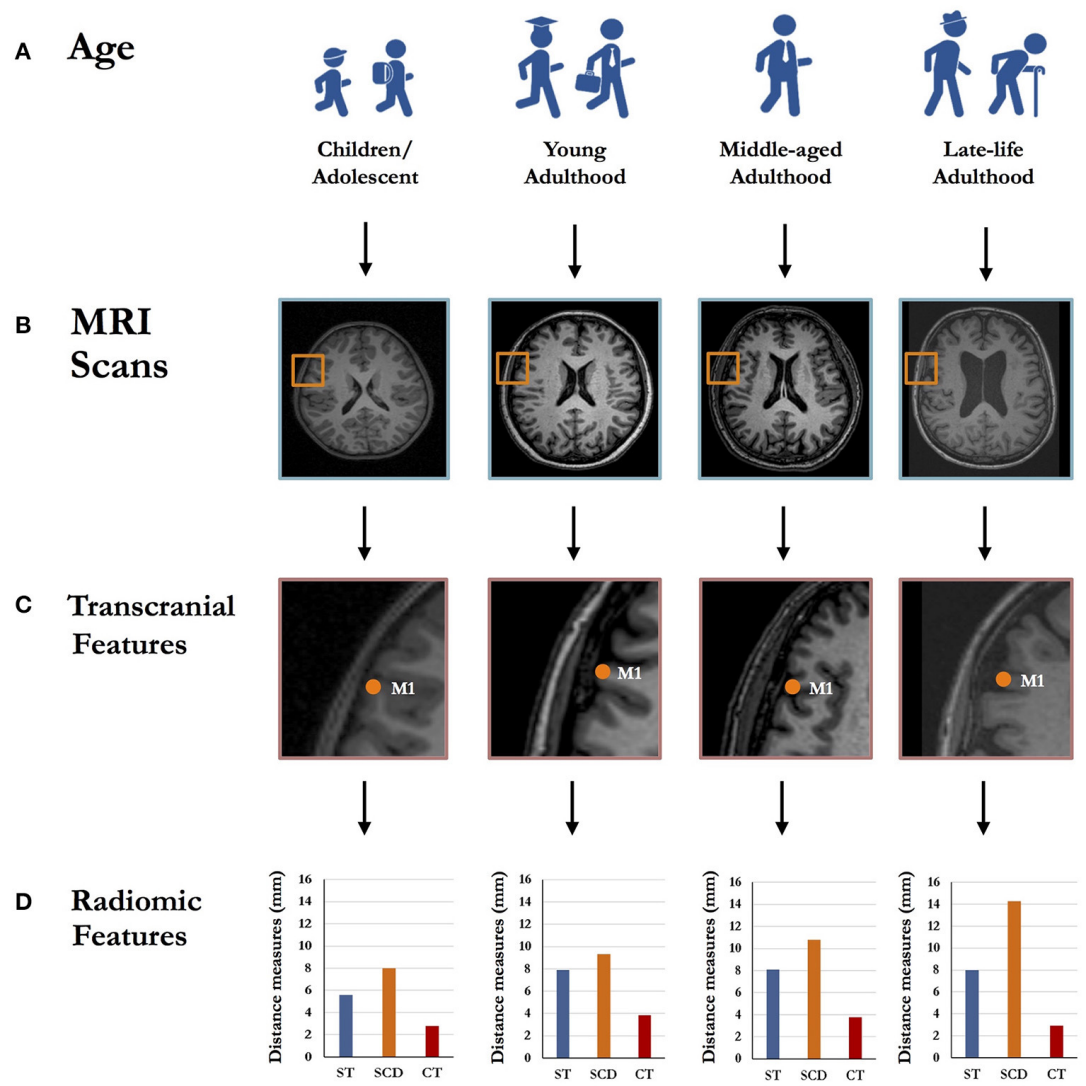
Schematic representation of the region-specific radiomic features differences across the individuals with different age ranges, including children/adolescent, young adulthood, middle-aged adulthood and late-life adulthood **(A)**. Taking primary motor cortex (M1) as an example, left M1 was localized by the Montreal Neurological Institute (MNI) coordinates through T1-weighted structural magnetic resonance imaging (MRI) scans **(B)**. The radiomic features in transcranial model **(C)**, including skull thickness, scalp-to-cortex distance and cortical thickness, vary across the individuals with different age ranges **(D)**. ST, Skull thickness; SCD, Scalp-to-cortex distance; CT, Cortical thickness.

As the most commonly used target in TUS studies (Kim et al., 2021), primary motor cortex (M1) was selected to demonstrate the transcranial features in individuals at different stages of life (Figure 1). With the same measurement scale (i.e., mm), the skull thickness, scalp-to-cortex distance and cortical thickness varied between children, young adult, middle-aged adult and old adult (Figure 1D). Compared to young and middle-aged adults, old adult showed increased scalp-to-cortex distance and reduced cortical thickness; while, children had thinner skull and cortex. Indeed, this simplified demonstration may have very limited power to quantify the brain features at the population level, but could highlight the possible heterogeneity in the transcranial and radiomic features of M1 among individuals at different stages of life. Based on prior evidence, the scalp-to-cortex distance of M1, rapidly increased during pathological aging (Lu et al., 2019a), plays as a determining factor in the dosimetry of TMS treatment (Stokes et al., 2005). Returning to skull thickness, evidence confirms a continuous increase in skull thickness and density during childhood and adolescence (Delye et al., 2015). Although the skull thickness slowly decreased during aging, significant changes were only found in female populations (Lillie et al., 2016), which address another interesting topic for future research of the gender effect on radiomic features. Toward delivering personalized TUS treatment in clinical practice, critical questions persist regarding the scalable features of the reconstructed scalp, skull and cortex that are based on transcranial model of the identification of the borders between non-brain tissues and brain parenchyma. Interestingly, pre-treatment transcranial model could be employed as a turnkey solution for optimizing the parameters for treatment targets and, meanwhile, examining the biophysical mechanisms of TUS at individual level. Previous studies focused on investigating the spatial distribution of TUS effect are based on the magnetic resonance imaging (MRI) or computed tomography scans of young adults with an average head size (Koh et al., 2021; Zhang et al., 2021). However, this standard model has limited power to represent the distribution of TUS-induced effects in the individuals with various skull and brain morphology. Therefore, the transcranial model of TUS should be developed in the combination of the features capturing the skull and cortex related to the stimulation targets. Besides, the discrepancies of region-specific radiomics may also rise the in advanced brain stimulation settings, such as multisite TUS.

## The Importance of Radiomic Features in TUS

High-resolution structural MRI provides a powerful modality in medical imaging, with the additional benefits of its high spatial resolution and plausible measurements of skull and brain morphology (Wahlund, 2020). Radiomics is a quantitative mapping approach to structural MRI, aiming at analyzing, extracting and quantifying the high-throughput features of human brain available to clinicians (Lambin et al., 2017). MRI-based radiomics, as a rapidly developing field, is an encouraging tool for the identification and quantification of region-specific brain features (Bretzner et al., 2021), which has been successfully applied in clinical practice, such as brain tumor (Zhang et al., 2019) and stroke (Chen et al., 2021). To improve the TUS protocols in age-related brain diseases, except for skull thickness, another transcranial variable, scalp-to-cortex distance (SCD), in the combination of cortical thickness, should be added into the head models for the individuals with different age ranges. Beside of global brain changes, region-specific brain morphometry plays a critical role in determining the precise localizations of targets (Weise et al., 2022) and predicting the disease progress (Lu et al., 2021) and neurophysiological outcomes (Mosayebi-Samani et al., 2021) in brain stimulation studies. Moreover, the dissociable trajectories of cortical thickness and SCD of M1 in old adults and dementia patients (Lu et al., 2019a) may capture the further assumptions about radiomic feature-dependent effect on focal in TUS studies.

As shown in Figure 1, compared to children, young and middle-aged adult, old adult showed a sharp change in skull thickness, SCD and cortical thickness. Due to the discrepancies of transcranial features, the importance of combining region-specific radiomic features in transcranial model for TUS treatment can be explained as follows: (1) Penetration depth: SCD is a vector-like parameter that serves as the distance measure connecting the point on the scalp to the point on the cortical surface (Lu et al., 2019b). SCD represents the sum of skull thickness and the thickness of connective tissues and CSF in two-dimensional space, but also represents other complex radiomic features in three-dimensional space, such as shape and curvature (Lu et al., 2021). Different layers of SCD have been identified with diverse conductivities that largely determine the penetration depth and its related dosage and effect of TUS. (2) Scaling-up neuron modeling: Regarding to the biophysics mechanisms of TUS, the range of TUS parameters for achieving efficient stimulation in terms of minimal acoustic intensity and energy deposition to the non-brain tissues and brain parenchyma has been mentioned in the transcranial model. To achieve the desired therapeutic effect, an activation of mechanosensitive ion channels induced by acoustic waves is the key part that ultrasound propagation travels through the non-brain tissues and cortical layers (Kamimura et al., 2020). Besides, the neuronal bilayer sonophore (NBLS) model has been developed for explaining the acoustic effect on the cell membrane and synapses of specific neurons at microscopic scale (Plaksin et al., 2016; Weinreb and Moses, 2022). It should be noted that cortical thickness represents an average of the distance from the inner surface of gyrus to the closest point on the outer surface of gyrus, containing six layers of neurons featured with cytoarchitectonic subdivisions (Amunts et al., 2013). According to the models of brain diseases, the interneurons embedded in specific layers are the targets related to neuropsychiatric symptoms or domain-specific cognitive function. Importantly, high-resolution MRI and ultrasound stimulation have comparable resolutions, which lie in the order of millimeter and can provide the spatial resolutions ranging from 1 to 2 mm^3^ (Wahlund, 2020). Therefore, the combination of scale-dependent MRI-based radiomic features (i.e., mm), SCD and cortical thickness in particular, may be critical to accurately modulate the activities of layer-specific neurons in TUS treatment.

To sum up, the dynamic changes of radiomic features are evident across the lifespan, whereas the scalp-to-cortex distance is increased linearly with age, accompanied by the non-linear changes of the thickness of skull and cortex. Given the complexity of transcranial structures, the region-specific radiomics derived from high-resolution structural MRI have created intriguing and encouraging opportunities for the personalization of transcranial brain stimulation in real-world clinical practice.

## Future Directions

With the guidance of individual head models, the TUS-mediated image-guided brain stimulation could be utilized in the implementation and demonstration of the precise and personalized modality with exquisite ability to deliver acoustic energy to the targeted regions. The therapeutic applications of imaging-guided TUS and its potential remedies in the context of transcranial mapping are future landscapes in neurosurgery and neurorehabilitation for the targeted drug delivery and early-stage disease modification. Beside of therapeutic utilities, image-guided TUS could also be used as a non-invasive and powerful tool for directly assessing and monitoring the function of the white matter tracts and cortical-subcortical networks *in vivo* at mesoscopic scale.

## Author Contributions

The author confirms being the sole contributor of this work and has approved it for publication.

## Funding

This study was supported by the Direct Grants for Research the Chinese University of Hong Kong (Project Numbers: 4054495 and 4054569).

## Conflict of Interest

The author declares that the research was conducted in the absence of any commercial or financial relationships that could be construed as a potential conflict of interest.

## Publisher's Note

All claims expressed in this article are solely those of the authors and do not necessarily represent those of their affiliated organizations, or those of the publisher, the editors and the reviewers. Any product that may be evaluated in this article, or claim that may be made by its manufacturer, is not guaranteed or endorsed by the publisher.
